# Ocular Emergencies in Children: Demographics, Origin, Symptoms, and Most Frequent Diagnoses

**DOI:** 10.1155/2020/6820454

**Published:** 2020-10-01

**Authors:** Henríquez-Recine, S. Noval, B. Zafra, S. De Manuel, I. Contreras

**Affiliations:** ^1^Department of Ophthalmology, La Paz University Hospital, IdiPaz, Madrid, Spain; ^2^Department of Ophthalmology, Ramón y Cajal University Hospital, Irycis, Rementería Clinic, Madrid, Spain

## Abstract

**Purpose:**

To describe the epidemiology of ocular emergencies in children in a hospital-based emergency room (ER).

**Methods:**

The medical reports of all children, 14 years of age and younger, who attended La Paz University Hospital (Madrid, Spain) ER from September 2015 to August 2016 were prospectively collected. Demographic data, origin, symptom for consultation, diagnostic tests, final diagnosis, and final referral of patients were recorded.

**Results:**

A total of 774 children were attended: 57% boys and 43% girls. Mean age was 5 years (SD 4.10 years, range 0–14 years) without significant differences between sexes. Most children went to the ER because parents or teachers took the decision (75%), 24% were referred from the paediatric ER for evaluation, and 0.78% were referred from another hospital or by an out-clinic ophthalmologist. The reasons for consultation were red eyes (61%), traumatism (17%), referred from the paediatrician to have the fundus explored in children with headache (7%), eyelids problems (7%), and visual loss (7%). The most frequent diagnoses were infectious conjunctivitis (29%), corneal erosion (17%), normal examination (15%), and allergic conjunctivitis (13%). The most severe cases (visual loss, acute strabismus, and leucocoria) were referred by the paediatricians and represented 4.65% of the total patients. Visits occurred more frequently during the third trimester of the year (July–September), with a higher incidence of eye trauma in summer.

**Conclusions:**

Infectious conjunctivitis was the most frequent pathology. It is necessary to increase public awareness about the symptoms, the way of transmission, and treatment. Ocular trauma was also a common cause of presentation and the majority were contusional and mild. There is also a need for education of parents, teachers, and coaches regarding the potential for eye injuries. Ophthalmologists and paediatricians must take an active role in educating people about the prophylactic measures to prevent eye injuries in children.

## 1. Introduction

Eye-related complaints are a frequent reason for consultation at any age and represent approximately 1–6% of the patients referred to the general emergency room (ER) around the world [1]. The incidence of ocular emergencies requiring urgent referral to the Emergency Department (ED) in the United States has been estimated at a national average of approximately 3 per 1,000 persons a year, with 2.4 million ocular injuries occurring every year [2]. The impact on adults is considerable because ocular injuries make up 3.3% of all occupational injuries resulting in lost workdays according to National Safety Council, 2002. One common cause for consultation is ocular trauma; it is the major cause of unilateral blindness in the United States, affecting 40,000 and 60,000 patients annually [3, 4].

Ocular emergencies and trauma are also prevalent in childhood. In a study at Wills Eye Hospital, eye emergencies treated within one year were evaluated and children represented 9% of all patients [5]. Most ophthalmological emergencies are mild, although sometimes they may correspond to pathology that threatens vision or constitute the first manifestation of diseases of the central nervous system. Timely diagnosis and referral by primary care providers are essential for successful outcomes in this small but clinically significant proportion of children [6].

Examining a child with a visual or ocular complaint can be a challenge at any moment. However, ocular complaints can include pain and visual impairment which may lead to anxiety and interfere with the examination of the child.

Very few studies have assessed the role of ER in providing eye care to children. Most existing epidemiological studies focus exclusively on traumatic emergencies [5]. The objective of this study is to determine the most frequent reasons for consultation and ophthalmological diagnosis in the ER in children. This will lead us to evaluate factors associated with visits and establish diagnostic and treatment protocols for the most frequent pathologies.

## 2. Materials and Methods

The medical reports of all children (range: 0–14 years of age) who attended La Paz University Hospital (Madrid, Spain) ER from September 2015 to August 2016 were prospectively recorded. We obtained the informed consent from the parents to collect the medical reports. The following demographic data were collected for every patient: age, gender, symptom for consultation, and origin. Data about ophthalmologic examination were also recorded when available: visual acuity (VA), evaluated with Snellen and Pigassou chart optotypes, pupillary reactions, extrinsic eye movements (using Hirschberg test, Cover test, and the broad *H* test, asking the patient to move the eyes in an “*H*” pattern, using an object like a pen or a toy and tracing an “*H*” in front of the patients while instructing them to hold their head still), biomicroscopy, intraocular pressure (IOP, Goldmann applanation tonometer), fundoscopy, and ancillary examinations, as well as final diagnosis, final destination, and the need for following examinations in the Paediatric Ophthalmology Department.

The data were analysed using IBM SPSS Statistics Data Editor (version 20). Chi square tests (Chi2), Fisher's exact test, and ANOVA test were used to study the statistical significance between the groups according to whether they were qualitative or quantitative variables. Student's *t* test was used to determine the significance of the difference between the means. We considered the existence of a statistically significant association between the variables under study when *p* <0.05.

## 3. Results

A total of 53,549 children were treated in the emergency room of the La Paz University Hospital between September 2015 and August 2016, 774 of them were ophthalmological emergencies, representing 1.45%, 441 boys (57%) and 333 girls (43%, *p* <0.001) from newborns to 14 year olds. Mean age was 5 years (SD 4.10 years) without significant differences between sexes (girls 5 years (SD 4.05 years) and boys 5 years (SD 4.14 years), *p* = 0.374).

Most children went to the ER because parents or teachers took the decision (75%), 24% were referred from the paediatric ED for evaluation, and 0.78% were referred from another hospital or by an out-clinic ophthalmologist.

**Figure 1:**
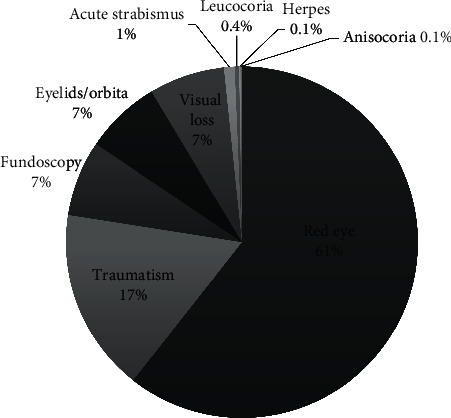
Reasons for consultation.

Figure 1 shows the reasons for consultation. The two most common causes were red eyes (61%) and traumatism (17%); 7% of children were referred, mostly from the paediatrician (92%), to have the fundus explored in children with headache; 7% went with eyelids or orbital problems such as cellulitis and 7% complaining of visual loss.

Table 1 summarises mean age, sex, and the most frequent referral source for each reason for consultation. Reasons for consultation such as visual loss, trauma, or referred fundoscopy were more common causes in older than the mean age children, while the youngest children were those referred due to leucocoria (*p* < 0.001, ANOVA test).

The role of paediatricians is essential in eye care, since the most severe cases were referred by them like those with visual loss, acute strabismus, leucocoria, or headache (the main reason for fundoscopy).

**Figure 2:**
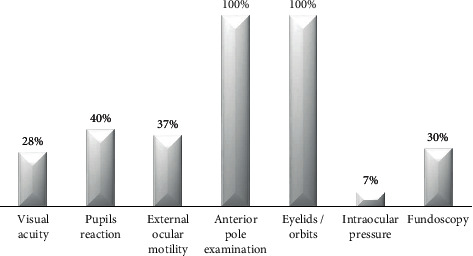
Ophthalmic examinations performed in children.

The ophthalmic examination was performed depending on the reason for consultation and the ability to carry it out according to age and collaboration (Figure 2). We evaluated the eyelids, the orbits, and the anterior biomicroscopy in 100% of the cases. In patients referring blurred vision, decrease in visual acuity, diplopia, traumatism, or suspected preseptal/orbital cellulitis, we studied the external ocular mobility.

**Figure 3:**
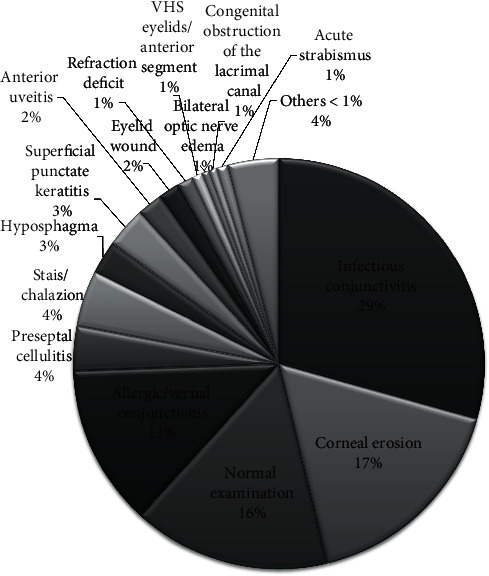
Most common final diagnosis.

The most frequent diagnoses were infectious conjunctivitis (29%) (4 of them with additional corneal erosion), corneal erosion (17%), normal examination (15%), and allergic/vernal conjunctivitis (13%).  Figure 3 shows the most common final diagnosis. Less than 1% of children were diagnosed of the more severe pathologies, which can be divided into those of traumatic cause, caused by accident, and not traumatic. Among the traumatic pathologies, we observed traumatic anterior uveitis (10 children), commotio retinae (3), retinal tear (3), hyphema (2), canalicular lacerations (2), and hemovitreous (1) and corneal perforation (2). These were more frequent in boys (85%) than in girls (15%), *p* = 0.007.

In the nontraumatic pathologies group, we observed anterior uveitis (7 children), optic disc oedema (5, 3 of them with idiopathic intracranial hypertension), glaucoma (4), orbital tumor (4), cataract (3), retinal detachment (2) (one of them presented with cataract and glaucoma), oculomotor nerve palsies (2), eyelid ptosis (1), facial palsy (1), and mucocele (1); 60% were boys and 40% girls, without significant differences between sexes (*p* = 0.765).

Ancillary tests were requested for 21 patients (3%) and carried out the same day, mainly image studies. Computed tomography (CT) was performed in 10 children; magnetic resonance imaging (MRI) in 4 children (one of them had both); ocular ultrasound in one child, and X-rays of the sinuses in another one. Optical coherence tomography (OCT) was performed in 4 children, cycloplegic refraction in 2, and blood testing in another 2. Cycloplegic refraction was only performed in the ER in cooperating children with acute strabismus. In other cases, if necessary, they were referred to the Paediatric Ophthalmology Department the following day.

**Figure 4:**
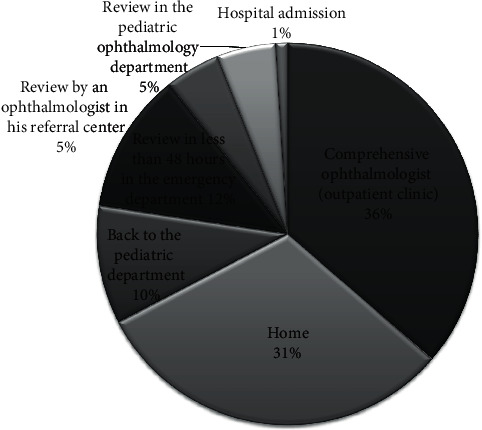
Final destination of children after evaluation in the ER.

Figure 4 shows where the children were referred once treatment, when necessary, was prescribed. Only 6 patients (0.78%) were admitted to hospital in charge of the Paediatric Ophthalmology Department, one for surgery and five for intravenous treatment. The five children admitted for intravenous treatment had an orbital cellulitis and were assessed by a multidisciplinary group of specialists in infectious diseases, paediatricians, otolaryngologists, and ophthalmologists. Antibiogram and culture were performed only in cases complicated with abscesses when the surgical sample was obtained.

**Figure 5:**
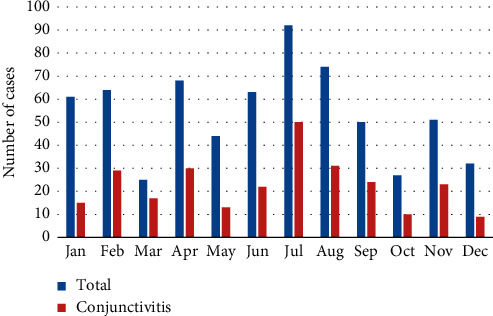
Conjunctivitis distribution throughout the year.

**Figure 6:**
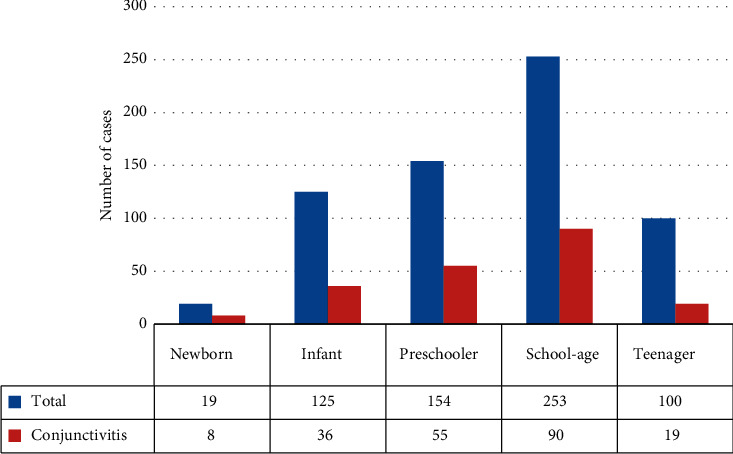
Age distribution for conjunctivitis.

Visits occurred more frequently during the third trimester of the year (July–September), with a higher incidence of eye trauma in summer. Figures 5 and 6 show the conjunctivitis distribution throughout the year and according to age, respectively, being more frequent in July and in children of preschool and school age.

**Figure 7:**
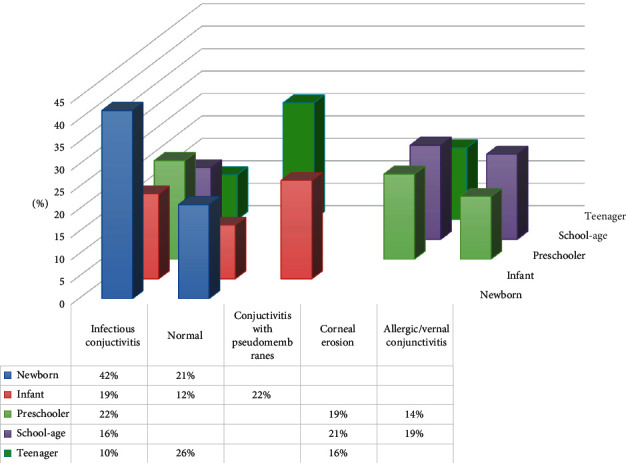
Distribution of the most frequent diagnoses according to age.

Considering the most frequent diagnoses according to age, infectious conjunctivitis was more frequent in newborns and preschool children, corneal erosions and allergic conjunctivitis in school-age children, and conjunctivitis with pseudomembranes in infants (Figure 7).

## 4. Discussion

La Paz University Hospital provides 24-hour care for acute eye problems over a population of approximately 787,000 inhabitants of the northern area of Madrid. The eye ER functions with one specialist doctor, covering 24 hours of duty and two or more trainees (resident doctors). The medical team also covers the hospitalised patients and consultations requested by other specialists. Out-clinic ophthalmologists attend the less severe cases and follow-ups from the ER.

There are few studies that analyse the epidemiology and characteristics of ophthalmologic emergencies in a general hospital, most of them make reference to traumatic injuries. Out of the total of 12,102 patients seen in one year, ocular emergencies in children represented the 6.4%. Cheung et al. studied hospital-based ocular emergencies for 5 hospitals of the United States, children and adolescents constituted 10.9% of the total patients [3].

Ophthalmological emergencies can be divided into those of traumatic cause, caused by accident, and not traumatic. In the current study, the prevalence of ocular trauma was 17%, being more frequent in boys (61%). Sánchez Tocino et al. published a prevalence of 13% of traumatic causes in all ages [7] and Macewen et al. published in their analysis a percentage of traumatic injuries of 38% [8]. In the study by Nelson et al. performed in the Wills Eye Hospital, eye injuries accounted for 38% of the total children population, they occurred two times more frequently in boys than in girls with no age prevalence [5]. They observed that during the second quarter of the year (April to June), the highest prevalence of ocular trauma to children occurred were 34%. In our study, ocular emergencies including trauma were more frequent in summer.

Red eye was the most frequent reason for consultation and infectious conjunctivitis the most frequent diagnosis. Previous studies [6, 9–11] have shown that an overwhelming proportion of ER visits were for red and watery eyes. Most cases of red eye were due to infectious conjunctivitis, which may be isolated or part of an outbreak [6].

The most common conjunctivitis overall in children are known to be noninfectious. In newborns, bacterial conjunctivitis are more frequent. Throughout the first year of life, bacterial conjunctivitis associated with lacrimal duct stenosis is also common, occurring in 5% of newborns although up to 90% resolve spontaneously. In children and adolescents, it is not clear whether viral (especially adenoviral) or bacterial conjunctivitis are more common [12]. In the study by Gigliotti et al., samples from children with conjunctivitis were cultured, observing growth of *Haemophilus influenzae* (42%) *Streptococcus pneumoniae* (12%), and adenoviruses (20%) as the three most frequent agents [13]. It is considered that in the group of conjunctivitis in childhood, only 10% have a bacterial origin. However, among the infectious ones, it is estimated that 50–75% are bacterial, although the presence of usual ocular flora questions these estimates [12].

Although the most common conjunctivitis are noninfectious, many cases of lacrimal duct stenosis and bacterial conjunctivitis in newborns, and allergic in children, are treated by paediatricians and allergists in primary care. Therefore, in our sample of patients, we mainly diagnosed infectious conjunctivitis, which was more frequent from April to August and was the most prevalent pathology in newborns (42%), infants (41%), preschoolers (36%), and school children (36%). Ovidiu et al. observed that inflammatory diseases were more prevalent in 54% of newborns, 80% of preschoolers, and 57% of scholars, being conjunctivitis the most frequent diagnosis. Among the newborns, 54% were diagnosed with conjunctivitis and 27% with lacrimal duct stenosis [14]. Their study showed a peak in conjunctivitis cases in late spring and late summer (May and August) seeing twice the cases presented in October. Other studies have also shown a constant increase of conjunctivitis cases during summer [14]. It is estimated that approximately 3 million school days are lost annually in the United States alone as a result of acute conjunctivitis, mostly viral in nature. School administrators and teachers should know how to prevent the spread of conjunctivitis in the classroom [6].

Paediatricians diagnose and treat many children with pathologies such as infectious and allergic conjunctivitis and lacrimal duct stenosis who do not attend to the ER. In our study, we observe the fundamental role they play in referring patients at risk of suffering from serious ophthalmological pathologies such as acute strabismus, leucocoria (which may be intraocular tumors or congenital cataracts), and headaches in patients with idiopathic intracranial hypertension and other neurological pathologies.

Our study has several limitations. We included patients attended by the ophthalmologist at the ER; however, there are known patients that may go directly to the Paediatric Ophthalmology Department. Besides, the La Paz University Hospital serves a large population for subspecialty care but may not be fully representative of the larger Madrid population. A longer study may be performed to draw more conclusions about severe pathology.

Infectious conjunctivitis was the most frequent pathology. It is necessary to increase public awareness and to educate teachers and parents about the symptoms, the way of transmission, and treatment. Ocular trauma was also a common cause of presentation to the ER and the majority were contusional and mild with very few cases of uveitis, hiphema, vitreous hemorrhage, and retinal tear. There is also a need for education of parents, teachers, and coaches regarding the potential for eye injuries. Ophthalmologists and paediatricians must take an active role in educating people about the prophylactic measures to prevent eye injuries in children.

## Figures and Tables

**Table 1:** Age, sex, and referral source for each reason for consultation.

Reason for consultation	Mean age	Most frequent sex	Most frequent source
Red eye	5 y-o (SD 3.83)	59% boys	Family or school (90%)
Traumatism	7 y-o (SD 3.77)	61% boys	Family or school (75%)
Fundoscopy	7 y-o (SD 4.74)	51% boys	Paediatrician (93%)
Eyelids or orbita	4 y-o (SD 3.94)	59% girls	Family or school (67%)
Visual loss	8 y-o (SD 4.31)	52% boys	Paediatrician (61.5%)
Acute strabismus	5 y-o (SD 3.68)	80% girls	Paediatrician (80%)
Leucocoria	4 months (0.33 y-o (SD 0.58))	67% boys	2 from the paediatrician

## Data Availability

The data from their research are available at their hospital's Statistics Department.
